# Molecular dynamic insight into octahydro-1,3,5,7-tetranitro-1,3,5,7-tetrazocine (HMX) and the nano-HMX decomposition mechanism

**DOI:** 10.1039/d2ra05394b

**Published:** 2022-11-14

**Authors:** Mingming Zhou, Genwang Wei, Yao Zhang, Dong Xiang, Caichao Ye

**Affiliations:** College of Chemistry and Environmental Engineering, Yangtze University Jingzhou Hubei 434023 PR China xiangdong@yangtzeu.edu.cn; Academy for Advanced Interdisciplinary Studies & Department of Physics, Southern University of Science and Technology Shenzhen Guangdong 518055 PR China yecc@sustech.edu.cn; Department of Materials Science and Engineering, Guangdong Provincial Key Laboratory of Computational Science and Material Design, Southern University of Science and Technology Shenzhen Guangdong 518055 PR China

## Abstract

Herein, we demonstrate the use of large-scale reactive molecular dynamics simulations to identify the influence of nanostructures, size effects, and temperature for the decomposition processes of octahydro-1,3,5,7-tetranitro-1,3,5,7-tetrazocine (HMX). The bulk-phase and six types of HMX nanoparticle (30–70 Å) systems were investigated at two high temperatures (2000 K and 3000 K). The evolution of the potential energy (PE) and total number of molecules (TM) of HMX crystals and their six nanoparticle systems were analyzed and addressed, and it was revealed that the nanostructure has a great accelerative effect on the thermal decomposition of HMX. The temperature distribution, initial decomposition process, and main intermediate and gas products were traced, and showed that the initial decomposition of HMX nanoparticles is triggered by the dissociation of the N–NO_2_ bond. With the increase in temperature, the total amount of gas molecules in HMX nanoparticles rapidly increases, which shows that the high temperature can accelerate the decomposition rate for HMX nanoparticles.

## Abstract 2

The entire decomposition reaction process of a 30 Å HMX nanoparticle at 2000 K by ReaxFF molecular dynamics.

## Graphical abstract

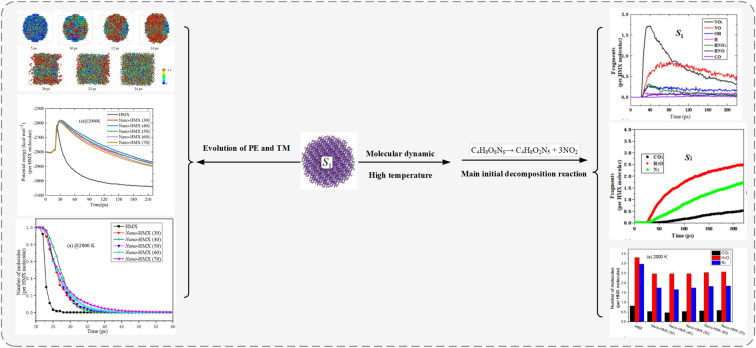

## 1. Introduction

Octahydro-1,3,5,7-tetranitro-1,3,5,7-tetrazocine (HMX) has been widely used as an explosive and rocket propellant, and its initial chemical processes have been investigated for many years. There has been a great deal of research over the years to investigate the initial chemical processes of HMX.^1–20^ There are two main factors that can affect the initial decomposition mechanism of HMX: (1) different external stimuli, and (2) the change in the explosive itself. Ge *et al.*^1,2^ studied the initial decomposition mechanism of HMX dependent on the impact velocity of shockwave loading. Sharia *et al.*^3^ commented on the revealed differences between solid and gas phase decomposition that proved that the crystalline field affects the bond-breaking processes in molecules, and molecular materials are strongly dependent upon the molecular environment. Defects and deformations play an important role^4,5^ in initiating chemical reactions in energetic materials by facilitating their degradation, and can consist of vacancies,^6–8^ crystalline polymorphs,^9–11^ dislocations,^12,13^ charged particles,^14,15^ and electronic excitations.^16,17*a,b*,18–20^

Nanoscale energetic materials can reduce the number of defects compared to conventionally sized energetic materials. The nano-structure may lead to high-shock initiation pressure^21^ and less sensitivity of the energetic materials.^22–28^ The combustion rate can be controlled by changing the particle size of nano-explosives.^29–33^ It has been proven that nanoHNS (2,2′,4,4′,6,6′-hexanitrostilbene) and nanoTATB are more sensitive than production grade explosives under a short duration pulse.^34,35^ The shock sensitivities of nano-sized HMX particle-contained samples are obviously lower due to the more regular particle surfaces. The detonation velocities of the nano-sized HMX particle-contained samples are higher, which is caused by the larger specific surface area of the nano-sized particles.^36^ The apparent activation energy as well as the thermal decomposition temperature of dense spherical nano-HMX particles were markedly decreased.^37^ Liu *et al.*^38^ studied the adiabatic initial decomposition processes of molecular explosive HMX nanoparticles with diameters of 1.4–2.8 nm at high temperatures from 2400 to 3000 K. Zhu *et al.*^39^ employed ReaxFF molecular dynamics (MD) to simulate the physicochemical properties accompanying some transitions of HMX nanoparticles at low temperatures. While nano-HMX has been studied for more than ten years, its pyrolysis mechanism at high temperature and closer to the actual nanoscale is unclear. Therefore, it is necessary to study the mechanism of pyrolysis at high temperature with a relatively larger nanoscale.

In this study, we demonstrate the use of large-scale reactive molecular dynamics simulations to identify the influence of nanostructures, size effects, and temperature for the initial decomposition mechanism of HMX. Herein, we simulated bulk-phase and six types of HMX nanoparticle (30–70 Å) systems at two high temperatures (2000 K and 3000 K). The initial decomposition pathways, the evolution of the small molecule products, and the evolution of the potential energy were investigated.

## 2. Computational details

**Fig. 1:**
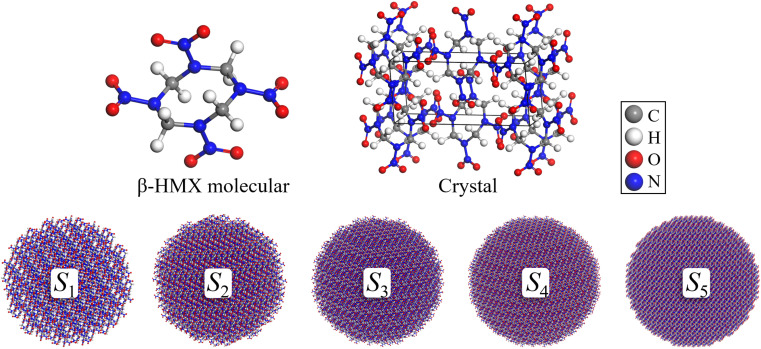
Structures of a single HMX molecule, crystal HMX, and HMX nanoparticles (30–70 Å). C, H, O, and N atoms are indicated by gray, white, red, and blue, respectively. These representations also apply to the following figures.

The LAMMPS molecular dynamics simulator was employed to perform simulations with ReaxFF-lg.^40^ An amorphous molecular model of nano-HMX was established with the radius of 30–70 Å. Each possible atomic pair was assigned a different bond order cutoff value to properly identify chemical species (standard values in ReaxFF C/H/O/N high-energy simulations).^41^ The original C/H/O/N ReaxFF has been used for several nitro explosives^41–44^ and nanoparticles^45,46^ and it correctly provided the 30–70 Å radius of nano-HMX, which was constructed from the original unit cell that was experimentally determined at room temperature.^47^ The initial state of the system is shown in Fig. 1, which contains 64 molecules (*S*_0_, 1792 atoms, 30.28 × 23.89 × 23.65 Å^3^), and other systems are shown in Table 1 with three-dimensional periodic boundary conditions.

**Table 1:** Detailed parameters of the six HMX nanoparticle models

System	Atoms	Molecules	Radius (Å)	Length of box (Å)
*S*_0_	1792	64	—	30.28 × 23.89 × 23.65
*S*_1_	12 488	446	30	64 × 64 × 64
*S*_2_	29 008	1036	40	86 × 86 × 86
*S*_3_	56 392	2014	50	106 × 106 × 106
*S*_4_	97 720	3490	60	126 × 126 × 126
*S*_5_	155 288	5546	70	146 × 146 × 146

First, we relaxed the nano-HMX by energy minimization with the conjugation gradient algorithm. To further relax the particles, with the canonical ensemble (NVT) and the Berendsen thermostat at 300 K, an MD simulation was performed for 10 ps. Then, we obtained the equilibrium nano-HMX crystal structure by an isothermal-isobaric (NPT) MD simulation at 0 atm and 300 K for 15 ps. Finally, the relaxed supercell was then used as the initial structure for six subsequent separate MD simulations. The relaxed supercell was heated from 300 to 2000 and 3000 K. Isothermal-isochoric MD (NVT-MD) simulations were then performed for 220 ps with the time step of 0.1 fs. An analysis of the products was performed with a 0.3 bond-order cutoff for all atom pairs to recognize molecular species. Within the minimum bond lifetime of 25 fs, if the distance between two atoms was less than the bond distance criterion, it was determined that the two atoms form a chemical bond.^48–52^ Therefore, the molecular species, bond orders, and dynamic trajectory were determined every 50 fs. These data were used to analyze the molecular species, and provided detailed information regarding the decomposition process.

The FORTRAN code FindMole^53^ based on the ideas of Strachan *et al.*^54^ was used to analyze the dynamic trajectories and calculate the chemical species and their numbers. The Getpath script was used to output net reactions and their frequencies.

## 3. Results and discussion

### 3.1. Evolution of the potential energy (PE) and total number of molecules (TM) in the HMX and nano-HMX system

**Fig. 2:**
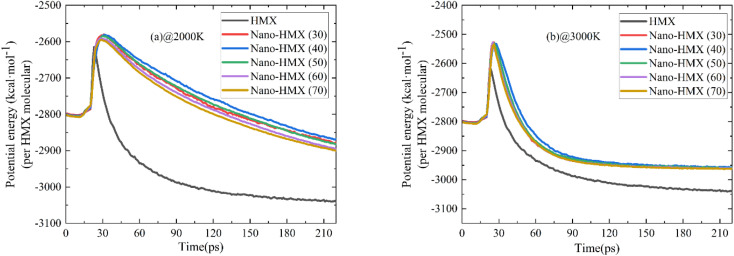
Evolution of the PE of the HMX and nano-HMX system with time at (a) 2000 K and (b) 3000 K.

Evolution of the PE and TM in the system at 2000 K and 3000 K within 220 ps is shown in Fig. 2. Initially, the curves rapidly increase and then decrease over time, indicating that energy is first absorbed and then released during decomposition. The PE curve of HMX reaches the maximum value earlier than that of nano-HMX, which indicated that it is necessary for nano-HMX to absorb more energy to achieve decomposition energy. As the temperature and nano-particle size increase, there is little change in the five PE curves. This demonstrates that the nanostructure greatly affects the PE curves, while there is little effect of nano-size and temperature on the PE curves. The PE curves of the HMX system reach stability at the maximum reaction time, which can be considered as completion of reaction. The PE curves of the HMX system decreased to equilibrium much more quickly as compared to the other nano-HMX system. The nano-size and temperature have little influence on the equilibrium time. There is a more rapid rate in the decrease of the PE curve, and the time to reach equilibrium is shorter for the HMX system. This indicates that the decomposition of nano-HMX is slower, and the reaction time is longer than that of the nano-HMX particles, while the nano-size and temperature have little effect on the stability of the HMX systems.

There are two turning points during the initial 30 ps of HMX thermolysis, which is interesting because the potential energy can roughly characterize the overall properties of the model system. The first turning point was at approximately 16 ps, and it may have been caused by the significant slippage in the surface layer at approximately 16 ps. This phenomenon is consistent with the conclusion in Fig. 4. The second turning point was at approximately 20 ps, and may have been caused by the rapid dispersion of the molecules and the approach of the initial decomposition reaction. At 30 ps, the maximum potential energy corresponded to all the nano-HMX molecules completely breaking down.

**Fig. 3:**
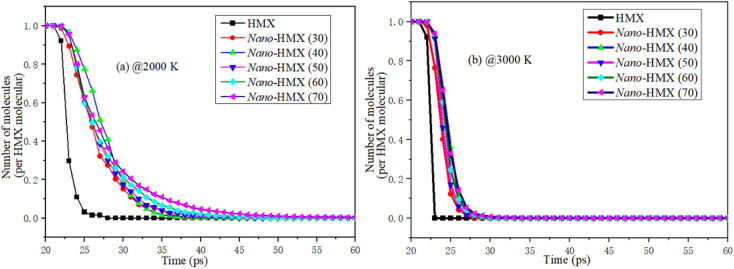
Evolution of the number of HMX molecules over time at (a) 2000 K and (b) 3000 K.

The number of molecules (NM) reflects the decomposition speed of the nano-HMX molecules. As Fig. 3 shows, the change in the NM in the system is similar at two high temperatures: first, the number of molecules remains stable for a period of the reaction time. Then, the number of molecules rapidly decreases. Finally, the number of molecules decreases during decomposition. When the temperature is higher, there is less time to decomposition, indicating that the reactions are more violent at higher temperatures. The decomposition rate increases with the increase in the nano-size at high temperatures.

**Fig. 4:**
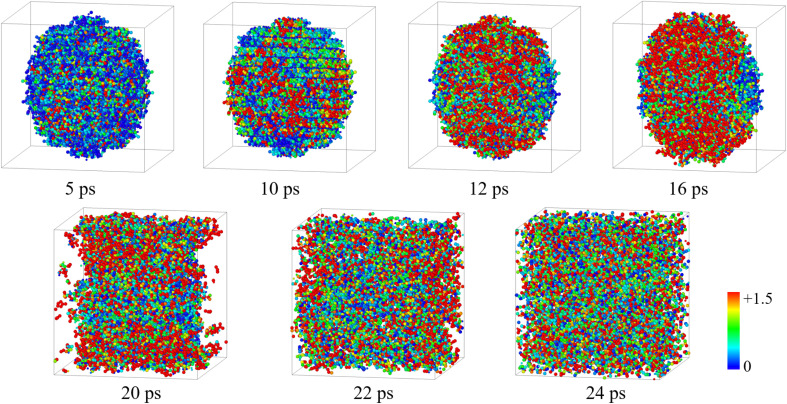
Temperature distribution of 30 Å nano-HMX for 0–24 ps at 3000 K.

Fig. 4 shows the temperature distribution of the 30 Å nano-HMX system for 0–24 ps at 3000 K. Due to the exothermic reaction, a few hot spots emerged at 5 ps (*T* > 1500 K). The diffusion of atoms is accelerated due to the atomic vibration conducting heat for further decomposition. The hot spots mainly occur in the center of the nano-ball (12–16 ps) and result in a microexplosion that leads to the ball falling apart (20 ps). Microexplosions promote the transfer of matter and energy to the gas phase (22–24 ps).

### 3.2. Decomposition reaction

**Fig. 5:**
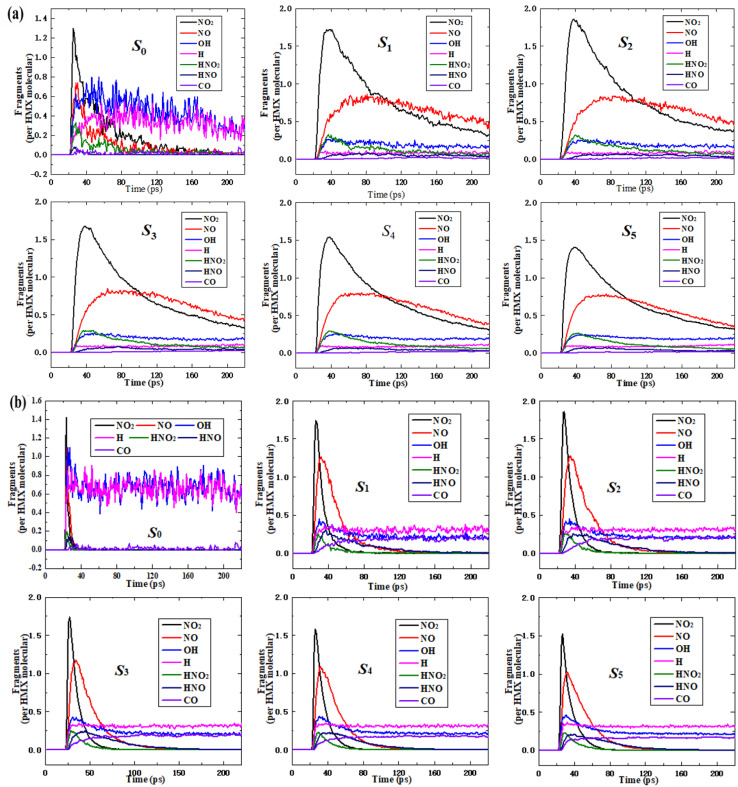
The main fragment analysis of the six systems at (a) 2000 K and (b) 3000 K.

The calculation results showed that the main fragments are NO_2_, NO, OH, H, HNO_2_, HNO, and CO in six systems at 2000 K and 3000 K, as shown in Fig. 5. All the curves of each fragment are similar for the nano-HMX system, while they are quite different from those of the HMX system. This demonstrates that the nanostructure strongly influences the main fragments. For all six systems, NO_2_ fragments initially appeared, and the number of NO_2_ molecules rapidly increased and then rapidly decreased. However, in the HMX system, the rate of decline was higher than that in the nano-HMX system. This demonstrates that NO_2_ decreased more quickly for the HMX system than that for the nano-HMX system. The NO_2_ totally decomposed for the HMX system, but for the nano-HMX system, there was less decrease in NO_2_, and it tended to be in equilibrium. The NO fragment rapidly increased and then reached the maximum value faster for the HMX system than that for the nano-HMX system.

The variation trend for other nitrogenous fragments such as HNO_2_ and HNO was consistent with that of the NO fragment for the six systems. All the nitrogenous fragments completely decomposed to produce more stable products such as N_2_ for the HMX system. However, they tended to be in equilibrium for the nano-HMX system, which demonstrates that the reaction for nano-HMX is much more complicated. The H fragment increased, while the H–R fragments decreased. Then, the H and OH fragments reached equilibrium. The number of OH fragments was larger than that of H fragments, which is abnormal.

To clarify the effect of temperature on the main intermediate products, an analysis was performed on the state of the major intermediate (Fig. 5(b)). The maximum of all the fragments was larger at 3000 K than that at 2000 K, while there was more rapid decomposition of all the nitrogenous fragments, which totally disappeared. This demonstrates that high temperature can accelerate the decomposition for the six systems. At 2000 K, there was little CO in the bulk HMX and nano-HMX systems. More CO was generated in the nano-HMX system than that for the bulk HMX system, which may have been caused by the nanostructure. At 3000 K, the amount of CO for the bulk HMX and nano-HMX systems increased, which may have occurred because heat increases the intensity of the pyrolysis reaction.

All the curves for CO_2_, H_2_O, and N_2_ of each fragment are similar to those of the nano-HMX system. However, they are quite different as compared to those for the HMX system. This demonstrates that the nanostructure strongly influences the main products. For the HMX system, the curves for CO_2_ and H_2_O initially increased, and then reached equilibrium. The curve for N_2_ rapidly increased, then slowly increased, and finally increased with rapidity once more. For the nano-HMX system, the curve for H_2_O increased, and then, there was a tendency to reach equilibrium. The curves for N_2_ and CO_2_ monotonically increased. For the six systems, the trend of the curve height is in the following order: H_2_O > N_2_ > CO_2_.

**Fig. 6:**
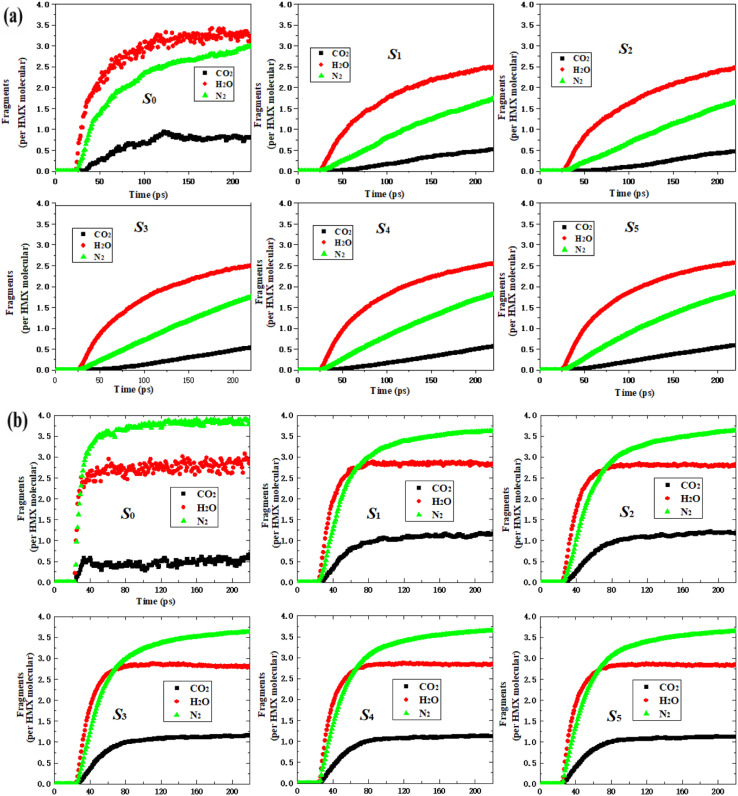
Time evolution of the products CO_2_, H_2_O, and N_2_ during the entire decomposition process of the HMX and nano-HMX systems at (a) 2000 K and (b) 3000 K. The thick trendline corresponds to the actual concentration data of the corresponding matching color.

To understand the effect of temperature on the main products, the main gas products were analyzed (Fig. 6(b)). The curves for three gas products indicate that less time is required to achieve a maximum value at 3000 K than that at 2000 K. For the six systems, the curves for three gas products initially increased, and then reached equilibrium. The equilibrium value of N_2_ was larger than that of H_2_O, while the equilibrium value of H_2_O was larger than that of CO_2_. The variation tendency for nano-HMX was similar, but the tendency for variation in the nano-HMX and HMX systems greatly differed. The increased rate of H_2_O production, the main product of nano-HMX, was much higher than that of N_2_ and CO_2_. Nevertheless, the trends for the growth rates of H_2_O and N_2_ were very similar.

**Fig. 7:**
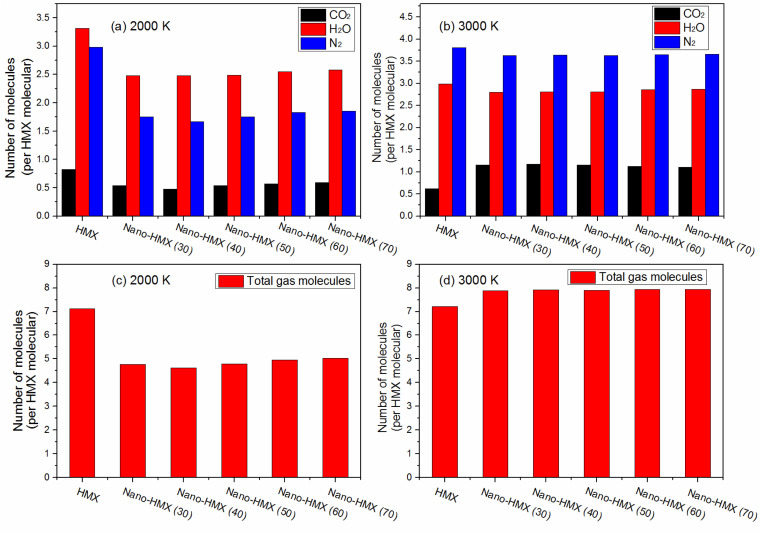
Time evolution of the products CO_2_, H_2_O, and N_2_ per HMX molecule during the entire decomposition process in the HMX and nano-HMX systems at (a) 2000 K and (b) 3000 K. Time evolution of the total gas molecules during the entire decomposition process in the HMX and nano-HMX systems at (c) 2000 K and (d) 3000 K.

As the temperature increased, the amount of CO_2_ and N_2_ increased, while the amount of H_2_O slightly decreased per HMX molecule for the nano-HMX system. However, as the temperature increased, CO_2_ and H_2_O decreased, while N_2_ increased per HMX molecule for the HMX system. The total gas molecules were almost the same at two high temperatures for the HMX system. However, the total gas molecules rapidly increased for the nano-HMX system as the temperature increased. These results indicate that high temperature can increase the specific impulse of the nano-HMX system, which further indicates that nano-HMX may be used as a propellant (Fig. 7).

The initial decomposition paths of the six HMX systems were analyzed by a MATLAB script. Table 2 shows the most frequent and important primary reactions of 30 Å nano-HMX at 3000 K. As shown in Table 2, at 0.2 ps, the C_4_H_8_O_8_N_8_ → C_4_H_8_O_2_N_5_ + 3NO_2_ reaction occurred at the highest frequency (23 times), and one HMX molecule decomposed and released three NO_2_ molecules. The C_4_H_8_O_8_N_8_ → C_4_H_8_O_4_N_6_ + 2NO_2_ reaction occurred 17 times at 0.2 ps, and one HMX molecule decomposed to release two NO_2_ molecules. At 0.2 ps, the C_4_H_8_O_8_N_8_ → C_4_H_8_N_4_ + 4NO_2_ reaction occurred 12 times. Combined with the result that NO_2_ appeared earlier than HNO_2_ in Fig. 5(b), the most likely initial reaction mechanism of nano-HMX is N–NO_2_ bond breakage and NO_2_ release.

**Table 2:** Primary reactions with the highest frequencies and the importance of 30 Å nano-HMX at 3000 K

No.	The initial reaction	Occurrence time (ps)	Frequency
R0	C_4_H_8_O_8_N_8_ → C_4_H_8_O_2_N_5_ + 3NO_2_	0.2	23
R1	C_4_H_8_O_8_N_8_ → C_4_H_8_O_4_N_6_ + 2NO_2_	0.2	17
R2	C_4_H_8_O_8_N_8_ → C_4_H_8_N_4_ + 4NO_2_	0.2	12
R3	C_4_H_8_O_8_N_8_ → C_4_H_7_O_2_N_5_ + NO_2_H + 2NO_2_	0.2	8
R4	C_4_H_8_O_8_N_8_ → C_4_H_7_N_4_ + NO_2_H + 3NO_2_	0.2	7
R5	C_4_H_8_O_8_N_8_ → C_4_H_8_O_6_N_7_ + NO_2_	0.2	2
R6	C_4_H_8_O_8_N_8_ → C_4_H_7_ON_5_ + OH + 3NO_2_	0.2	2
R7	C_4_H_8_O_8_N_8_ → C_4_H_7_O_4_N_6_ + NO_2_H + NO_2_	0.2	2
R8	2C_4_H_8_O_8_N_8_ → C_4_H_7_O_2_N_5_ + C_4_H_8_O_2_N_5_ + NO_2_H + 5NO_2_	0.2	2

This result indicates that the N–NO_2_ bond is decomposed in nano-HMX. This phenomenon is consistent with the result that LEWIS used BLYP and B3LYP to explore the decomposition path of HMX in the gas phase. LEWIS found that the reaction energy barrier of N–NO_2_ cleavage (40.5–41.8 kcal mol^−1^) was the lowest among four possible paths (HONO elimination, C–N bond breaking, and joint ring opening); that is, N–NO_2_ cleavage was the most likely cleavage path of HMX. The most frequent of the three reactions was the release of multiple NO_2_ molecules from a nano-HMX molecule, which adequately explains the rapid growth rate of NO_2_ after the initial reaction in Fig. 5. At 0.2 ps, the C_4_H_8_O_8_N_8_ → C_4_H_7_ON_5_ + OH + 3NO_2_ reaction occurred twice. The appearance of OH adequately explains the phenomenon that OH appears earlier than H in Fig. 5, while the HNO_2_ fragment in R3, R4, R7, and R8 adequately explains the phenomenon that the amount of OH is greater than H in Fig. 5.

One of the results reported by the software is that the net flux (NF) indicates how often the reaction is observed during the simulation time. Table 3 demonstrates the relatively higher NF of the reactions that have relatively higher NF at 2000 K and 3000 K.

**Table 3:** Elementary reactions and net flux (NF) of 30 Å nano-HMX at 3000 K

No.	Reactions	NF	No.	Reactions	NF
R0	CO_2_N_2_ → CO_2_ + N_2_	5321	R31	CN_3_ + N_2_ → CN_5_	762
R1	CO_2_ + N_2_ → CO_2_N_2_	5276	R32	CO + ON → CO_2_N	757
R2	CON_2_ → CO + N_2_	3527	R33	CHON_3_ → CHON + N_2_	684
R3	CO + N_2_ → CON_2_	3477	R34	CHON + N_2_ → CHON_3_	669
R4	ON + N_2_ → ON_3_	3214	R35	ON + HON → ON + HON	664
R5	ON_3_ → ON + N_2_	3206	R36	HO + ON → HO_2_N	659
R6	N_4_ → 2N_2_	2953	R37	CO_2_N → CO + ON	629
R7	2N_2_ → N_4_	2933	R38	H_2_O_2_N → ON + H_2_O	563
R8	CON + N_2_ → CON_3_	2623	R39	CHN_2_ + N_2_ → CHN_4_	563
R9	CON_3_ → CON + N_2_	2620	R40	CHN_4_ → CHN_2_ + N_2_	554
R10	2ON → O_2_N_2_	1493	R41	HN_2_ + N_2_ → HN_4_	545
R11	HN_2_ + N_2_ → HN_2_ + N_2_	1443	R42	HN_4_ → HN_2_ + N_2_	537
R12	O_2_N_2_ → 2ON	1399	R43	ON + H_2_O → H_2_O_2_N	534
R13	H_2_ON_2_ → H_2_O + N_2_	1155	R44	HO + HON → ON + H_2_O	525
R14	H_2_O + N_2_ → H_2_ON_2_	1154	R45	C_2_N_3_ + N_2_ → C_2_N_5_	503
R15	HO + N_2_ → HON_2_	1126	R46	ON + H_2_O → HO + HON	497
R16	ON + CON → CO_2_N_2_	1119	R47	C_2_N_5_ → C_2_N_3_ + N_2_	490
R17	HON_2_ → HO + N_2_	1075	R48	HO + H_2_O → HO + H_2_O	477
R18	C_2_O_2_N → CO + CON	1074	R49	H_2_N_3_ → H_2_N + N_2_	469
R19	CO_3_N → CO_2_ + ON	1066	R50	H_2_N + N_2_ → H_2_N_3_	456
R20	CO_2_N_2_ → ON + CON	1050	R51	H + N_2_ → HN_2_	395
R21	CO + CON → C_2_O_2_N	1013	R52	HN_2_ → H + N_2_	394
R22	CO_2_ + ON → CO_3_N	1010	R53	CHO_2_N_2_ → CHON + ON	386
R23	ON + HON → HO_2_N_2_	937	R54	H_2_N_2_ → H_2_ + N_2_	384
R24	HO_2_N_2_ → ON + HON	927	R55	CHON + ON → CHO_2_N_2_	380
R25	C_2_O_3_ → CO + CO_2_	854	R56	C_2_ON_4_ → C_2_ON_2_ + N_2_	378
R26	CO + CO_2_ → C_2_O_3_	844	R57	C_2_ON_2_ + N_2_ → C_2_ON_4_	367
R27	HON_3_ → HON + N_2_	841	R58	CN_3_ → CN + N_2_	361
R28	HON + N_2_ → HON_3_	822	R59	H_2_ + N_2_ → H_2_N_2_	352
R29	HO_2_N → HO + ON	786	R60	CHO_2_N_2_ → HN_2_ + CO_2_	351
R30	CN_5_ → CN_3_ + N_2_	767	R61	C_2_ON_4_ → C_2_N_3_ + ON	349

One of the major intermediate species is R-N_2_ for the maximum net flux. The important reactions that release N_2_ occur due to the high concentration of N_2_, and this molecule appears at (R0, 2, 5, 6, 9, 11, 13, 17, 27, 30, 33, 42, 49, 52, 54, 56, 58). Another major intermediate is R-CO_2_ for releasing CO_2_, and this molecule appears at (R0, 25, 60). The overall analysis of simulations shows that the other main product gas is CO_2_. The other main intermediate is R-H_2_O to release H_2_O, and this molecule appears at (R38, 44, 48). The chemical effect of H_2_O can be expressed in terms of two types of reactions: H_2_O_2_N → ON + H_2_O (R38) and HO + HON → ON + H_2_O (R44).

## 4. Conclusions

In this work, the decomposition mechanism of octahydro-1,3,5,7-tetranitro-1,3,5,7-tetrazocine (HMX) and nano-HMX by size effect and temperature was studied by ReaxFF-lg reactive MD. The results illustrate the detailed mechanism and morphological evolution of HMX and its nanospheres. The main conclusions are as follows:

(1) For the variation rate in the PE curve of HMX and nanoparticles with different sizes at different temperatures, it was found that the nanostructure greatly influences the PE curve. However, there is little influence of the nanosize and temperature on the PE curve.

(2) By comparing the change trend of TM in the six systems at different high temperatures, it was found that the larger the nano-size, the higher the reaction temperature, and the more intense the reaction.

(3) By analyzing the temperature distribution of nano-HMX, it was found that the heat conduction process of the entire reaction was endothermic, heat transfer, pyrolysis and heat release, and the corresponding morphological change of nano-spheres was vibration-sphere dissociation and diffusion.

(4) For the variation in the main intermediate fragments and products, it was found that the nanostructure and high temperature have a strong influence on the main fragments, while there is little influence of nanosize on the pyrolysis reaction.

(5) At 3000 K, the initial decomposition reaction of 30 Å nano-HMX is C_4_H_8_O_8_N_8_ → C_4_H_8_O_2_N_5_ + 3NO_2_, which occurs at a maximum frequency of 23 times at 0.2 ps. The most likely initial reaction mechanism of nano-HMX is N–NO_2_ bond breaking and NO_2_ release.

We expect that these theoretical studies of the reaction of HMX might stimulate further experimental studies of nanoparticle-HMX, particularly the synthesis and characterization.

## Conflicts of interest

There are no conflicts to declare.

## Supplementary Material
